# Single-cell sequencing resolves the landscape of immune cells and regulatory mechanisms in HIV-infected immune non-responders

**DOI:** 10.1038/s41419-022-05225-6

**Published:** 2022-10-04

**Authors:** Haiyu Li, Yongyao Tang, Yujing Wang, Yue Li, Yi Yang, Kui Liao, Fangzhou Song, Shixiong Deng, Yaokai Chen

**Affiliations:** 1 Department of Infectious Disease, Chongqing Public Health Medical Center, 400036 Chongqing, China; 2 Chongqing Institute of Green and Intelligent Technology, Chinese Academy of Sciences, 400714 Chongqing, China; 3 School of Medical Information, Chongqing Medical University, 400016 Chongqing, China; 4 Basic Medical College, Chongqing Medical University, 400016 Chongqing, China; 5 The First Clinical College of Chongqing Medical University, Chongqing Medical University, 400016 Chongqing, China; 6 Department of radiotherapy, The First Affiliated Hospital of Chongqing Medical University, 400016 Chongqing, China

**Keywords:** HIPPO signalling, HIV infections

## Abstract

Immune non-responder after highly active antiretroviral therapy (HAART) is the main cause of opportunistic infections and high mortality in AIDS patients, but the mechanism underlying immune reconstitution failure is poorly understood. Here, we performed scRNA-seq, and scATAC-seq analysis of peripheral blood mononuclear cells (PBMCs) derived from immune non-responder (INR) and responder (IR) HIV-1-infected subjects. We found low expression of mucosal-associated invariant T (MAIT) cells in INRs, which exhibited transcriptional profiles associated with impaired mitochondrial function and apoptosis signaling. Single-cell assays for transposase-accessible chromatin (scATAC-seq) and flow cytometry revealed diminished mitochondrial fitness in MAIT cells from INRs, and MAIT had low expression of transcription factor A for mitochondria (TFAM) and peroxisomal proliferator-activated receptor alpha (PPARA). These findings demonstrate that restoring mitochondrial function could modulate the immune dysfunction characteristic of MAIT against bacterial co-infections in INRs subjects.

## Introduction

Acquired immune deficiency syndrome (AIDS) is a fatal infectious disease caused by the human immunodeficiency virus (HIV). The main pathological feature is a significant reduction in CD4^+^ lymphocyte count, resulting in a series of immunodeficiency-related diseases. An increasing body of evidence suggests that combined antiretroviral therapy (cART) can significantly reduce viral replication in HIV/AIDS patients, increase the CD4^+^T lymphocyte count, reconstitute the immune function and reduce mortality [1–5]. However, there are still 15–20% of AIDS patients with complete inhibition of virus replication after cART, and the CD4^+^ T-cell count cannot be restored to the normal level of uninfected people, a phenomenon termed immune non-response (INR) [6–10]. INR increases the susceptibility to opportunistic infections or non-AIDS-related diseases, such as cardiovascular, liver and kidney disease, leading to higher mortality rates than immune responders (IRs) with restored CD4^+^T-cell count [11–15]. Opportunistic infections are common in INR patients, and the incidence rate and mortality are also high. In addition to AIDS-related diseases and deaths, non-AIDS-related diseases and deaths are also higher in nonimmune responders [16–19]. Current evidence suggests that low CD4^+^T-cell counts increase the incidence rate and mortality of cardiovascular diseases and are closely related to the occurrence of AIDS-related or non-related tumors and HIV-related neurocognitive diseases.

To better understand the mechanism underlying immune reconstitution failure, we used single-cell RNA sequencing (scRNA-seq) and single-cell assay for transposase-accessible chromatin with high-throughput sequencing (scATAC-seq) to examine the single-cell transcriptional profile of nonimmune and immune responders. We found lower mucosal-associated invariant T cells (MAIT) expression in INRs, which displayed transcriptional profiles associated with impaired mitochondrial function and apoptosis signaling. MAITs are part of the immune system; their main task is to control bacteria on the body barrier (such as skin and mucosa). MAIT cells represent unconventional T cells with the dual characteristics of innate and adaptive immunity. They can be activated in a TCR-dependent or independent manner [20, 21]. They respond quickly to stimuli and can affect the response of T cells and B cells in the early stage of immune response [22–26]. This study explores the molecular mechanism of MAIT mitochondrial dysfunction in HIV-infected immune non-responders.

## Results

### Single-cell transcriptome atlas of PBMCs in IRs and INRs

**Fig. 1:**
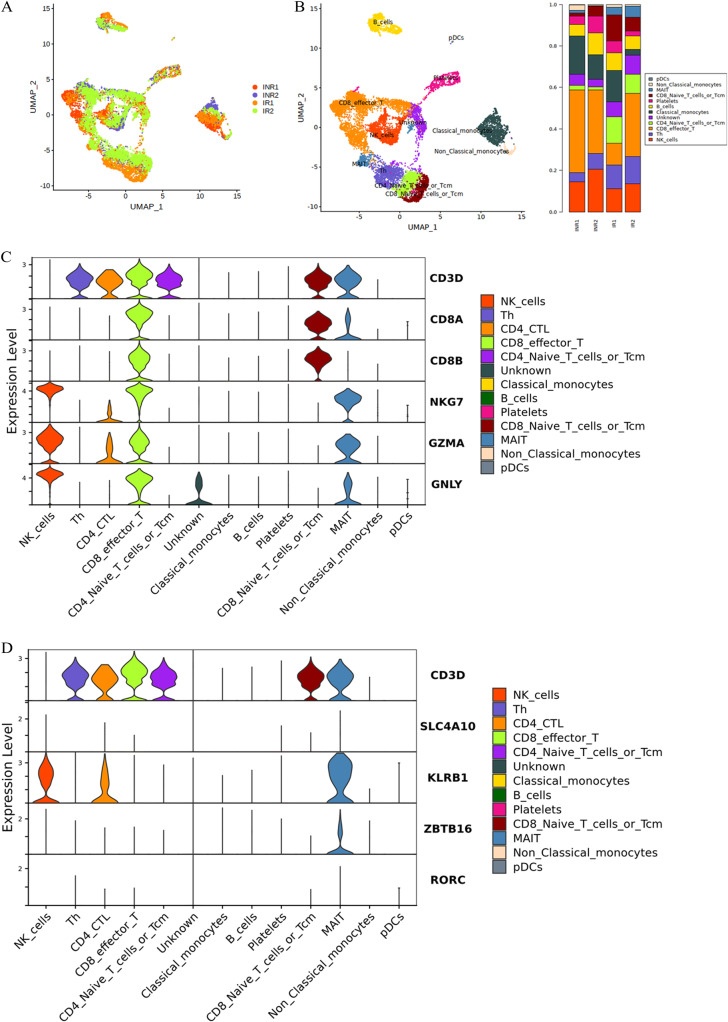
Single-cell transcriptional profiling of PBMCs derived from IR and INR patients with HIV-1-infected. **A** Two-dimensional UMAP visualization of PBMCs for IRs and INRs. Different colors represent different sample sources. **B** Different colors represent 10 clusters (cell types) defined by the *k*-means clustering algorithm. Different colors represent 11 cell types. The UMAP projection of 12086 single cells from IR and INR samples shows 11 clusters with the respective labels. Each dot corresponds to a single cell, colored according to cell type. **C**, **D** Stacked violin plots showing the expression distribution of selected canonical cell markers in the CD8 MAIT T cells.

To identify the immunological features of HIV-1-infected patients, we performed droplet-based single-cell RNA sequencing technology (10×Genomics) of fresh PBMCs derived from 2 immune non-responders (INR1 and INR2) and 2 immune responder controls (IR1 and IR2). After quality control and a unified single-cell analysis pipeline, the total number of recovered cells was 12086, comprising 5172 cells for INR and 6915 cells for controls IRs detecting a mean of 1,445 and 1,384 genes per cell, respectively (Fig. 1A, B and Fig. S1A). We calculated subsets that showed high differences between cells in the dataset (i.e., high and low expression in distinct cells). The screened differential genes with hypervariable characteristics were used for downstream principal component analysis. We visualized the cells in two-dimensional spaces according to their expression profiles using graph-based clustering of uniform manifold approximation and projection (UMAP), a nonlinear dimensionality reduction algorithm (Fig. 1A). Based on the expression of canonical gene markers [27], we identified cells to 11 major cell types or subtypes: CD8 effector T, NK cell, T helper, B cell, classical monocytes, nonclassical monocytes, pDCs, mucosal-associated invariant T cell, platelets, CD4 naïve T cells, and CD8 naïve T cells (Fig. 1B). A detailed list of marker genes defining cell subsets is provided in Supplementary Table 1 and Fig. S1B. To visualize single-cell RNA-Seq data, we constructed a website at http://150.158.212.80:8081. This website is open to the public and allows visualization of the expression of genes of interest in specific cell types without registration. These clusters and their specific markers included CD3D, CD8A, CD8B, and NKG7 (Fig. 1C, D).

### Significant reduction and dysfunction of MAIT cells in INRs

**Fig. 2:**
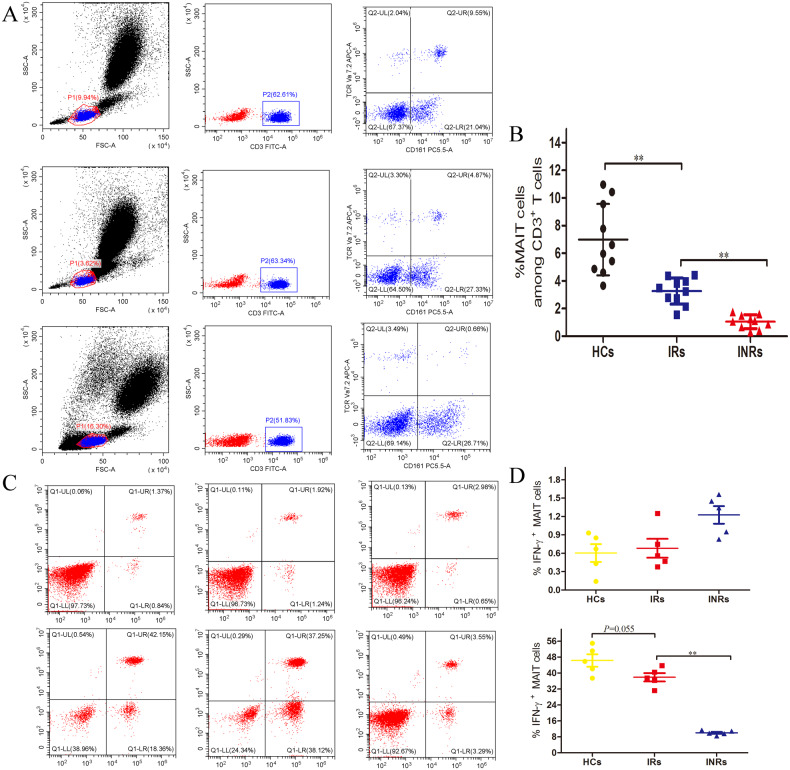
MAIT derived from INR is dysfunctional. **A**, **B** Proportions of MAIT cells detected in PBMCs from healthy controls (HC), immune non-responder (INR) HIV-1-infected subjects and immune responder (IR) HIV-1-infected subjects. Representative FC (flow cytometry) plots (**A**) and statistical analysis (**B**) are shown. **C**, **D** Detection of interferon-gamma produced by MAIT after PBMC isolated from HCs, INRs, and IRs was stimulated by paraformaldehyde (PFA)-fixed *E. coli*.

Single-cell transcriptome sequencing showed that the proportion of MAIT cells in INR decreased significantly. To assess the frequency of MAIT cells, FACS analyses were performed in peripheral blood mononuclear cells (PBMCs) from HIV-1–infected immune non-responders (*n* = 17), HIV-1–infected immune responders (*n* = 10), and uninfected healthy blood donors (*n* = 10). MAIT cells were identified using cell-surface markers CD3^+^ TCRV7.2^+^CD161^high^ in PBMCs (Fig. 2A). We found that the frequency of MAIT cells was significantly lower in INRs than in IRs or HCs (Fig. 2B). INRs were defined as having CD4^+^ T-cell counts below 350 cells/μl and IRs as having CD4 + T-cell counts above 350/μl after at least 2 years of cART with virologic control. The level of IFN gamma was detected in MAIT cells derived from IR, INR, and HC stimulated by paraformaldehyde (PFA)-fixed *E. coli*. MAIT cells from INRs produced lower levels of IFN gamma in response to *E. coli* stimulation than the HC group (Fig. 2C, D). These results suggest that MAIT derived from INR exhibited dysfunction in resisting infection by other pathogens.

### MAIT cells from INRs have distinct gene expression profiles reflecting apoptotic signaling and inflammatory responses

**Fig. 3:**
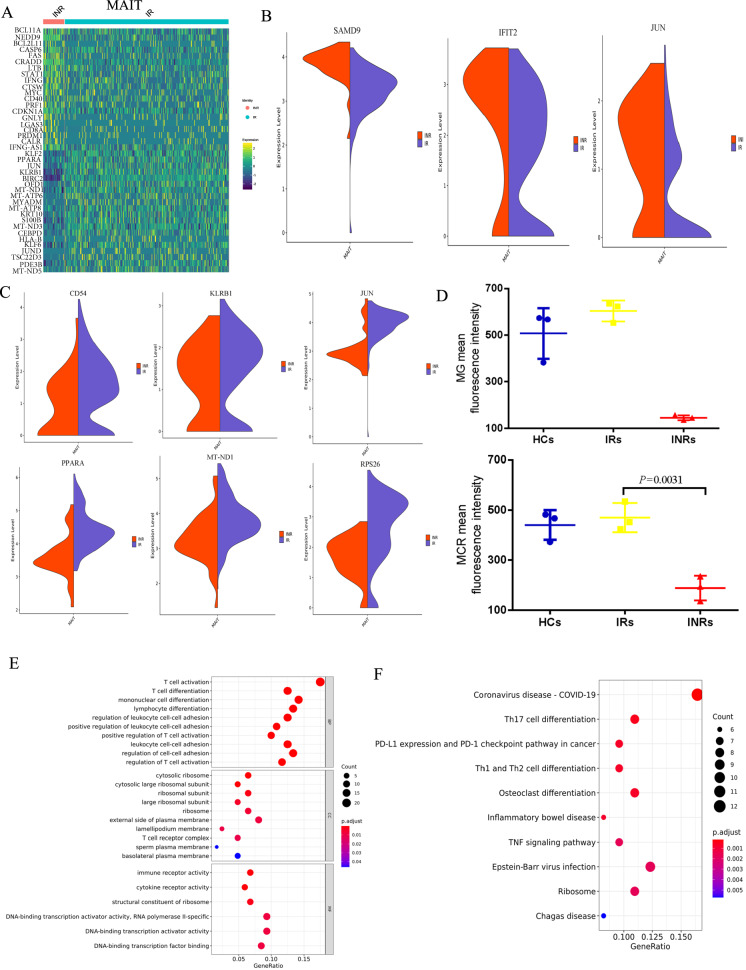
The apoptotic gene expression profile of MAIT cells derived from INR. **A** Heatmaps of differentially expressed genes in MAIT cells between IR and INR groups. **B** Violin plots of the proapoptotic gene expression in MAIT cells from INRs and IRs. **C** Violin plots of mitochondrial function-related gene expression in MAIT cells from INRs and IRs. **D** Mitochondrial mass and mitochondrial membrane potential are estimated by MG fluorescence and MRC fluorescence in MAIT cells from INRs and IRs, respectively. **E**, **F** GO and KEGG analyses conducted using DAVID for upregulated genes in MAIT cells from INRs compared with IRs.

To define the molecular mechanism of MAIT dysfunction in INRs, we next explored differences in gene expression profiles of MAIT cells between INRs and IRs. We found that the expression of proapoptotic genes in MAIT cells was significantly (*P* < 0.05) upregulated in INRs than in IRs (Fig. 3A). Furthermore, we found that the expression of proapoptotic genes IFI27, IFIT2, and SAMD9 was significantly increased in MAIT cells from INRs (Fig. 3B). As shown in Fig. 3C, the expression of *CD54*, *KLRB1*, and *JUN*, regulators of T-cell function and *MT-ND1*, *MT-ATP6* and *RPS26*, master regulators of mitochondrial biogenesis, were significantly decreased in MAIT cells from INRs. It is well-established that mitochondrial function is closely related to apoptosis [28–30]. We analyzed the transcriptional profile of mitochondrial-related genes (*MT-ND1*, *MT-ATP6* and *RPS26*) in INR and found decreased expression of genes involved in different phases of mitochondrial function of MAIT cells from INRs. To confirm whether the mitochondrial function of MAIT is impaired, Mito-Tracker Green (MG) and Mito-Tracker Red CMXRos (MRC) were used to detect mitochondrial mass and mitochondrial membrane potential, respectively (Fig. 3D). The oxidative phosphorylation activity measured by the mean fluorescence intensity of MCR was significantly decreased in MAIT cells derived from INRs. Similarly, when the mean fluorescence intensity of MG was used to measure the quality of mitochondria, the MFI significantly decreased in MAIT cells from INR (Fig. 3D). The Gene Ontology (GO) and KEGG enrichment analysis further showed that the upregulated genes in MAIT cells from INRs were significantly enriched in Type I IFN and apoptosis pathways (Fig. 3E, F).

### TFAM and PPARA low expression was associated with MAIT cell dysfunction of INRs

**Fig. 4:**
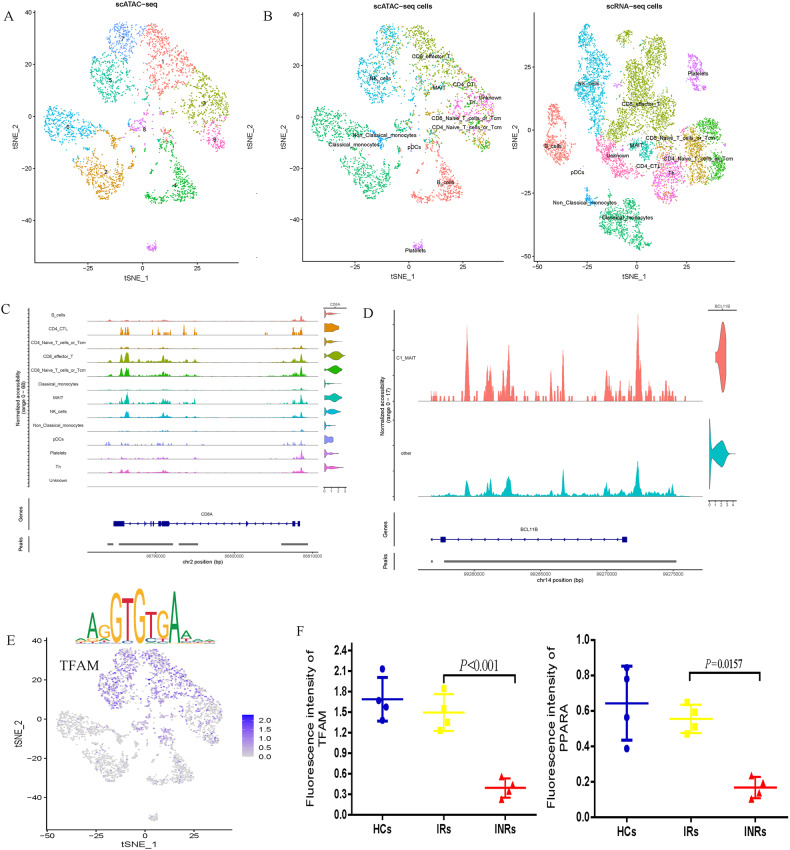
scATAC-Seq reveals open chromatin landscapes of single cells in INR. **A** tSNE plots showing single-cell open chromatin profiles analyzed in the study, color-coded for cell clusters. **B** Display after integration of scATAC-Seq and scRNA-seq. **C** scATAC-seq tracks showing open chromatin peaks associated with cell-type-specific genes across different cell clusters. **D** Chromatin open state of BCL11B gene in MAIT cells derived from INR. **E** Transcription factor motif enrichment (upper) and gene expression profiles (bottom row) for TFAM. **F** Mean fluorescence intensity of TFAM and PPARA expression in MAIT cells from HCs, IRs and INRs.

To identify the mechanism that drives MAIT cell dysfunction, we performed scATAC-Seq on PBMC derived from INRs. After normalization and dimensional reduction, we identified nine major clusters of cells, together with open chromatin peaks specific to each cluster (Fig. 4A). We integrated the scRNAseq and scATAC-seq datasets to correlate and cross-validate gene expression profiles and chromatin accessibility landscape in INR (Fig. 4B). To identify transcription factors (TFs) essential for cell-type-specific gene expression, we profiled TF binding motifs overrepresented in the peaks that showed differential accessibility among cell types (Fig. 4C and Fig. S2A). We also found that the chromatin domain of BCL11B was active and open in MAIT cells derived from INR, indicating that BCL11B may be highly expressed in INR-derived MAIT cells (Fig. 4D). Having established the high concordance between the scRNAseq and scATAC-seq data, we found that transcription factor A for mitochondria (TFAM) binding activity was significantly decreased in INR (Fig. 4E and Fig. S2B). Furthermore, the expression of TFAM and peroxisomal proliferator-activated receptor alpha (PPARA), the master regulator of mitochondrial biogenesis, were detected by flow cytometry. As shown in Fig. 4F, MAIT cells from INRs showed significantly decreased expression of PPARA and TFAM.

### Cytotoxic CD4 T cells are significantly reduced in INRs

**Fig. 5:**
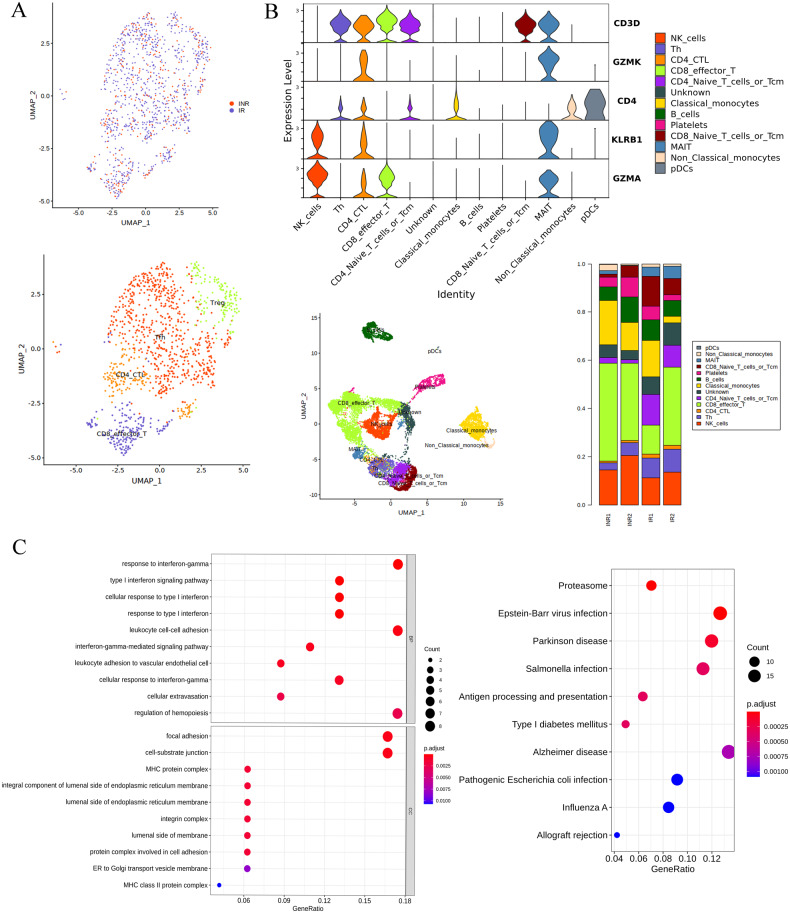
Profile of Th cells in INRs. **A** Two-dimensional UMAP visualization of Th cells using the Seurat R package. Different colors represent two groups (up). Different colors represent different cell subsets (down). **B** Expression and proportion of cytotoxic genes in CD4 CTL. **C** GO and KEGG analyses conducted using DAVID for upregulated genes in CD4 CTL cells from INRs compared with IRs.

Our single-cell analysis established that the proportion of Th cells in INR was significantly decreased. It is widely acknowledged that Th cells play an important role in the immune system, and their function is to produce a variety of cytokines, transmit antigen information, promote the differentiation and proliferation of T and B cells, and assist B cells in producing antibodies. Th cells were extracted from all cells to better understand the function of this Th cell-specific population in INR and further analyzed using the Seurat R software package. The Th cluster was divided into four small cell subsets using a clustering algorithm based on shared nearest neighbor modular optimization (Fig. 5A). Based on the expression of established marker genes, we identified a marked increase of cytotoxic CD4 T cells (CD4 cytotoxic T lymphocytes [CTLs]) in IR and INR groups (Fig. 5A). We mapped CD4 CTL to the two-dimensional UMAP of single-cell transcriptome and found that the proportion of CD4 CTL in INR was significantly reduced compared with IR (Fig. 5B). We then compared these 2 groups and identified 440 differentially expressed genes of CD4 CTL. GO analysis of highly expressed genes in CD4 CTL derived from INR showed significant enrichment in cell over-activation (Fig. 5B).

### The profile of CD8 effector T cells in INRs

**Fig. 6:**
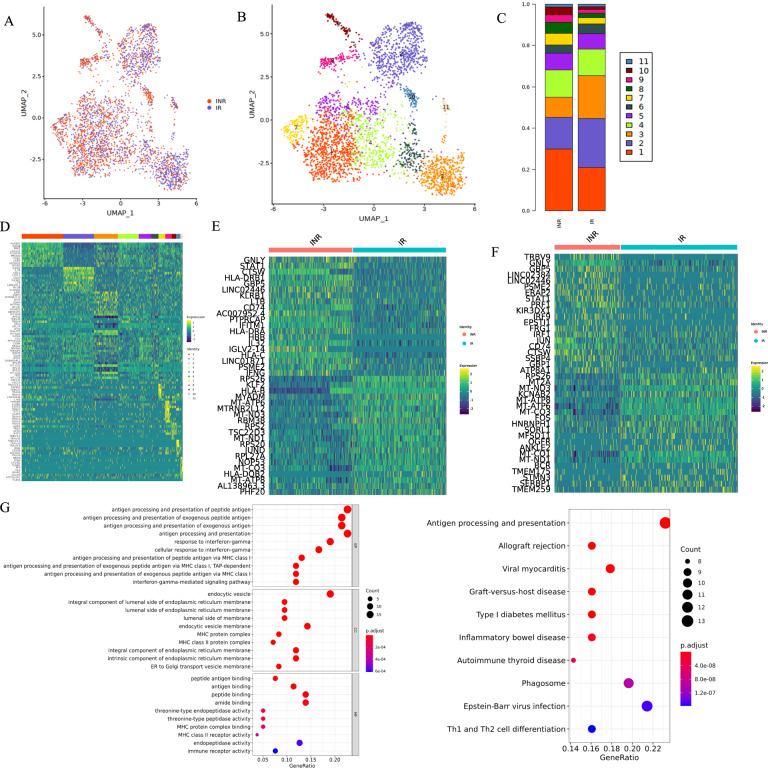
Profile of CD8 effector T cells in INRs. **A** UMAP visualization of CD8 effector T cells using the Seurat R package. Different colors represent two groups (left). Different colors represent different cell subsets (right) (**B**). **C** Proportion of CD8 effector T cells. **D** Differentially expressed genes in CD8 effector T cells between IRs and INRs ©. **E** Heatmap of top 40 genes significantly expressed in CD8 effector T C1. **F** Heatmap of top 40 genes expressed in CD8 effector T C2. **G** GO and KEGG were analyzed using DAVID for upregulated genes in CD4 CTL cells from INRs compared with IRs.

CD8 effector T cells are specific T cells that secrete various cytokines to participate in immune function and kill antigenic substances such as viruses and tumor cells. Our previous results showed no significant difference in the CD8 effector T-cell proportion between INR and IR. To better understand the function of CD8 effector T cells, we extracted all cells and subdivided them into 11 small cell clusters (C1–11) using the Seurat R package (Fig. 6A, B). The proportion of CD8 effector T cells C1 and C2 was significantly different in INR and IR, possibly due to different biological functions (Fig. 6C). We then compared these two groups and identified differentially expressed genes of CD8 effector T, CD8 effector T C1 and C2 (Fig. 6D–F). GO analysis of highly expressed genes in CD8 effector T C1 derived from INR showed significant enrichment in the type 1 interferon signaling pathway (Fig. 6G).

### Cell state transition of CD8 effector T cells, MAIT cells, and CD4 CTL during T-cell differentiation

**Fig. 7:**
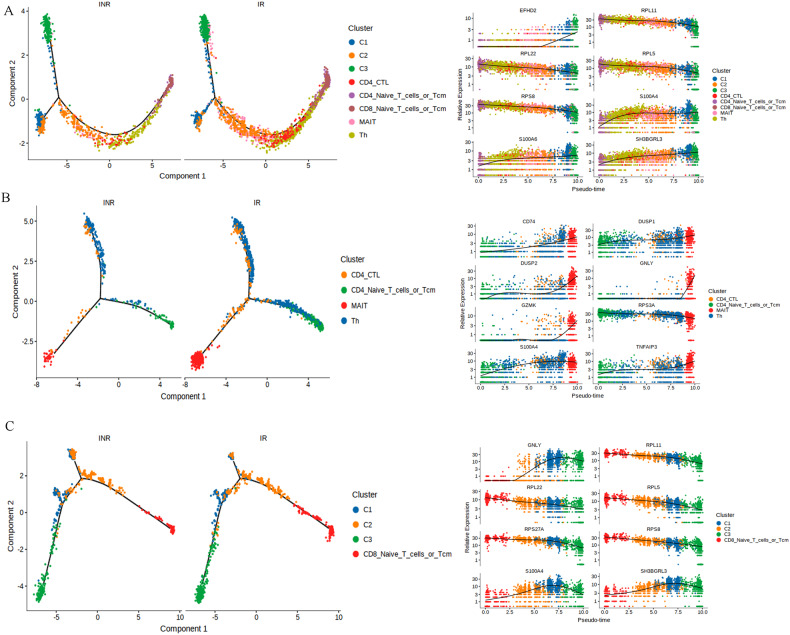
The differentiation state of T cells in INRs and IRs. **A** Pseudotime trajectory of T cells estimated using Monocle 2. A continuous value from 0 to 12 was assigned to each cell as a pseudotime. (Right) Expression transition of differentiation-associated genes along the pseudotime. **B** Pseudotime trajectory of CD4 CTL, MAIT, and Th cells estimated using Monocle 2. (Right) Expression transition of differentiation-associated genes along the pseudotime. **C** Pseudotime trajectory of CD8 effector T cells estimated using Monocle 2.

To understand the difference in T-cell differentiation between INRs and IRs, we constructed single-cell trajectories using the Monocle2 R package. According to the gene expression profile changes, all T cells, including CD8 effector T cells, MAIT cells and CD4 CTL, were placed on these trajectories (Fig. 7A). Consistent with the clustering analyses, there was continuity in the differentiation of MAIT and Th cells in IRs, while there was a gap in the differentiation trajectory in INRs (Fig. 7B). There was no significant difference in the differentiation trajectories of CD8 effector T cells in IRs and INRs (Fig. 7C).

### Identification of the cytokine profile for INR and IR

**Fig. 8:**
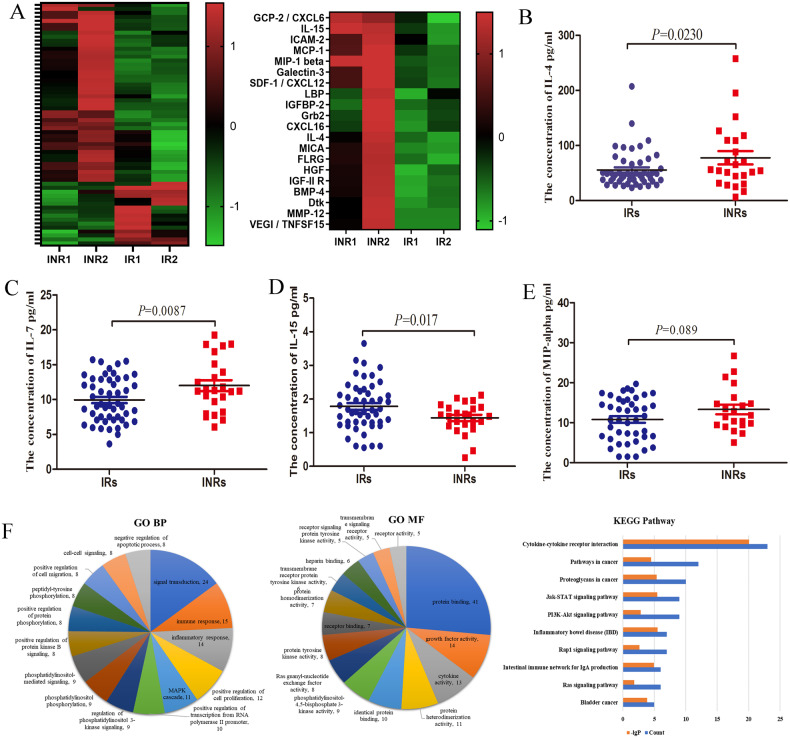
Cytokine profile between INR and IR. **A** Heatmap of significantly expressed cytokine between INRs and IRs using cytokine antibody microarray. **B**, **C**, **D**, and **E** Concentrations (pg/ml) of IL-4, IL-7, IL-15, and MCP-1 in the plasma samples obtained from INR and IR patients. **F** GO and KEGG analyses were conducted using DAVID for upregulated cytokine in plasma from INRs compared with IRs.

To screen differential cytokines between INR and IR, L-serious 507 antibody-based protein microarrays were performed to measure the inflammatory cytokine expression profile. Expression levels of 62 cytokines were significantly different between the INR and IR patients (*P* < 0.05). The top 20 cytokines with the most significant differences are shown in Fig. 8A. To verify the cytokine microarray results, a liquid-suspension cytokine microarray was performed to detect concentrations of IL-4, MCP-1, IL-7, and IL-15 in plasma samples from IRs (*n* = 51) and INRs (*n* = 24) (Fig. 8B–E). GO and KEGG enrichment analysis further showed that the upregulated cytokines in plasma from INR subjects were enriched in cellular activation pathways, including cytokine–cytokine receptor interaction, inflammatory bowel disease etc, indicating that INRs sustained abnormal immune activation (Fig. 8F).

## Discussion

Current evidence suggests that highly active antiretroviral therapy (HAART) can significantly reduce viral replication in HIV/AIDS patients, increase the CD4^+^ T lymphocyte count and reconstruct the immune function of patients [31]. However, some patients still experience immune reconstitution failure during viral inhibition [32]. Many factors are related to the failure of immune reconstitution in HIV/AIDS patients. Over the years, several retrospective studies have been conducted on patients with poor immune reconstitution. Overwhelming evidence substantiates that age, baseline CD4^+^ T lymphocyte level, HCV co-infection and other factors can affect the immune reconstitution of patients [33–36]. However, the mechanism underlying immune reconstitution failure is poorly understood. Single-cell sequencing technology is a new technology for high-throughput sequencing analysis of genome, transcriptome and epigenome at the single-cell level. It can reveal the gene structure and gene expression state of a single cell, reflect the heterogeneity between cells, and reveal the biological mechanism of disease.

In this study, we sequenced the single-cell transcriptome of PBMC from INR and IR patients. Cluster analysis identified 11 cell subsets, including CD8 effector T, NK cell, T helper cell, B cell, classical monocytes, nonclassical monocytes, pDCs, mucosal-associated invariant T cell, platelets, CD4 naïve T, and CD8 naïve T. Single-cell transcriptome sequencing and flow cytometry results confirmed that the proportion of MAIT cells in INR decreased significantly compared with IR subjects. We found that MAIT cells derived from INR were dysregulated under the stimulation of *E. coli* and could not produce an interferon response. MAIT cells derived from INR are in a state of depletion, resulting in dysfunction. About 5% of T cells in healthy subjects are composed of MAIT cells, mainly used to control bacteria, but the immune system can also recruit them to fight viral infections. The results of scATAC-seq and flow cytometry showed that the low expression of TFAM and PPARA predominantly accounted for mitochondrial dysfunction in MAIT cells derived from INR.

Furthermore, we identified a marked increase of cytotoxic CD4 T cells (CD4 cytotoxic T lymphocytes [CTLs]) in IR and INR subjects. In 1977, it was first reported that CD4 T cells cultured in vitro exhibited cytotoxicity. There are few studies on CD4 T cytotoxic cells, and much controversy surrounds the presence of CD4 T CTL in the human body. In 2001, sunI et al. reported that CD4^+^ CD8^-^T lymphocytes had strong cytokine expression and proliferation ability and showed cytotoxic activity when infected with HCV and HIV-1 virus. Since then, research on CD4 cytotoxic T cells has gradually attracted attention. In this study, we also found a cluster of CD4^+^ T lymphocytes with high specific expression of cytotoxicity-related genes, which further confirmed that there might be CD4^+^ CTL in the human body with potential cytotoxicity-related functions. Therefore, this cluster was defined as “CD4^+^ CTL”.

In summary, we revealed the single-cell expression profiles in INR and IR by scRNA-seq and scATAC and established that low expression of TFAM accounted for mitochondrial dysfunction in MAIT derived from INR and the poor clinical outcomes.

## Materials and methods

### Human blood samples

Human blood samples involved in this study were approved by the Ethics Committee of Chongqing Public Health Medical Center. Informed consent was obtained from all subjects. Immune non-responder and responder HIV-1 infected patients were recruited at the AIDS Outpatient Department of Geleshan Hospital of Chongqing Public Health Medical Center. The blood samples of healthy individuals came from Chongqing Public Health Medical Center (Pingdingshan hospital area). PBMCs were isolated from fresh whole blood in a 15 ml centrifuge tube using Ficoll-Paque PREMIUM (Cytiva, USA, 17544203) according to the manufacturer’s instructions. After mixing the blood sample with sterile PBS in equal volume, the mixture was slowly added to the Ficoll cell separation media and centrifuged 500 × *g* horizontally for 15 min at room temperature. Cell numbers and viability were measured using a hemocytometer with Trypan blue staining.

### scRNA-seq library construction

The single-cell library was constructed using the ChromiumTM Controller and Chromium Single Cell 5ʹ Library Kit. Briefly, single cells, reagents and Gel Beads containing barcoded oligonucleotides were encapsulated into nanoliter-sized GEMs using the GemCode Technology. Lysis and barcoded reverse transcription of polyadenylated mRNA from single cells was performed inside each GEM. Post-RT-GEMs were cleaned up, and cDNA was amplified. cDNA was then fragmented, fragment ends were repaired, and A-tailing was added to the 3’ end. The adaptors were ligated to fragments that were double-sided SPRI selected. Another double-sided SPRI selection was carried out after sample index PCR. The quality and quantity of the final library were validated using two methods: checking the distribution of different fragment sizes using the Agilent 2100 bioanalyzer and quantifying the library using real-time quantitative PCR. The final products were sequenced using the MGISEQ (BGI-Shenzhen, China).

### Single-cell data analysis

Cell Ranger Single Cell Software Suite (v3.1.0) was used to align complementary DNA reads to the reference genome. Single-cell FASTQ sequencing reads from each sample were processed and converted to digital gene expression matrices. The dataset was trimmed of cells with fewer than 200 genes. The number of genes, UMI counts and percentage of mitochondrial genes were examined to identify outliers. Principal component analysis was used for dimensionality reduction, followed by clustering in principal component analysis space using a graph-based clustering approach. UMAP was then used for two-dimensional visualization of the resulting clusters. For each cluster, the marker genes were identified using the FindConservedMarkers function implemented in the Seurat package (logFC. threshold > 0.25 and minPct > 0.25). Then, clusters were remarked to a known cell type according to Cell Marker database [27, 37, 38]. Differently expressed genes across different samples were identified using the FindConservedMarkers function in Seurat using the screening criteria: logFC. threshold > 0.25, minPct > 0.25, and adjusted *P*-value ≤0.05’. Pseudotime trajectory analysis was conducted with the R package Monocle2 [38].

### GO and KEGG analysis

GO and KEGG pathway analyses were performed using the phyper R function. Only GO terms or KEGG pathways with FDR ≤ 0.05 were significantly enriched. R package WGCNA was used to identify highly correlated genes using parameters “biweight midcorrelation > 0.7”. The highly correlated genes were used for the final correlation network construction.

### Antibodies and flow cytometry analysis

Fluorochrome-conjugated monoclonal antibodies specific for human antigens were used anti-CD4-FITC (BD Bioscience, catalog 561005), anti-TCR Vα7.2-APC (BioLegend, catalog 351708), anti-IFN-γ-APC (BD Bioscience, catalog 562017), Anti-CD3-FITC (Bioscience, catalog 555339), Anti-CD161-PE (BD Bioscience, catalog 556081). Intracellular staining was performed using eBioscience™ Intracellular Fixation & Permeabilization Buffer Set (ThermoFisher) according to the manufacturer’s protocols.

### Cytokine antibody microarray assay

The AAH-BLG-507 cytokine antibody microarray (Raybiotech, Norcross, GA, USA) was used to detect cytokines in plasma samples derived from IR and INR subjects. The detection of cytokines was performed according to the manufacturer’s instructions. Briefly, plasma was subjected to sample dialysis, sample labeling, antibody array blocking and incubation, and antibody array scanning detection. When the raw data were analyzed, the background removed fluorescent signal FI (F532 medium-B532 medium) was generally used for analysis, the mean value of two repetitions of all antibodies was calculated, and the mean value was used as the signal value of each antibody for subsequent analysis. All antibodies of each sample were normalized with all positive controls to obtain the normalized signal value. Generally, in the chip experiment results, for proteins with low signal value, a signal value FI < 25 was regarded as “not detected”.

### Luminex liquid-suspension microarray

The concentrations of selected cytokines in the plasma samples derived from 51 IR subjects and 24 INR subjects were detected using a human high sensitivity T-cell magnetic bead panel (Millipore) according to the manufacturer’s instructions.

The samples underwent incubation with magnetic beads, detection antibodies and Luminex200 detection. After the samples and standards tested in this experiment were detected by Luminex 200 detector, the fluorescence obtained was automatically detected and optimized by software to form the raw data. According to the fluorescence detection value obtained from the standard, the standard curve and its equation are obtained by fitting the standard curve with multi-parameter mode, and the concentration unit is pg/ml. The original fluorescence detected by each sample was substituted into the standard curve formula to calculate the sample concentration, used for comparison between samples.

### Statistical analysis

All data are expressed as means ± SD. Statistical analyses were carried out using GraphPad Prism 8.0 software. *P*-value was calculated by *t*-test or one-way analysis. *P*-value < 0.05 was considered significant.

## Supplementary information

Reproducibility checklist

supplementary

supplementary Table 1

## Data Availability

All datasets generated/analyzed for this study are included in this published article.
