# Correction: Single-cell sequencing resolves the landscape of immune cells and regulatory mechanisms in HIV-infected immune non-responders

**DOI:** 10.1038/s41419-023-05940-8

**Published:** 2023-07-12

**Authors:** Haiyu Li, Yongyao Tang, Yujing Wang, Yue Li, Yi Yang, Kui Liao, Fangzhou Song, Shixiong Deng, Yaokai Chen

**Affiliations:** 1 Department of Infectious Disease, Chongqing Public Health Medical Center, 400036 Chongqing, China; 2 Chongqing Institute of Green and Intelligent Technology, Chinese Academy of Sciences, 400714 Chongqing, China; 3 School of Medical Information, Chongqing Medical University, 400016 Chongqing, China; 4 Basic Medical College, Chongqing Medical University, 400016 Chongqing, China; 5 The First Clinical College of Chongqing Medical University, Chongqing Medical University, 400016 Chongqing, China; 6 Department of radiotherapy, The First Affiliated Hospital of Chongqing Medical University, 400016 Chongqing, China

**Keywords:** HIPPO signalling, HIV infections

Correction to: *Cell Death and Disease* 10.1038/s41419-022-05225-6, published online 04 October 2022

In this article were errors in the combination of images in Figure 1C and 1D, resulting in the two images being the same, which could lead to misunderstandings for readers. We request correction of the explanation and resubmission of Figure 1D. But this correction will not affect the conclusion of the entire study.
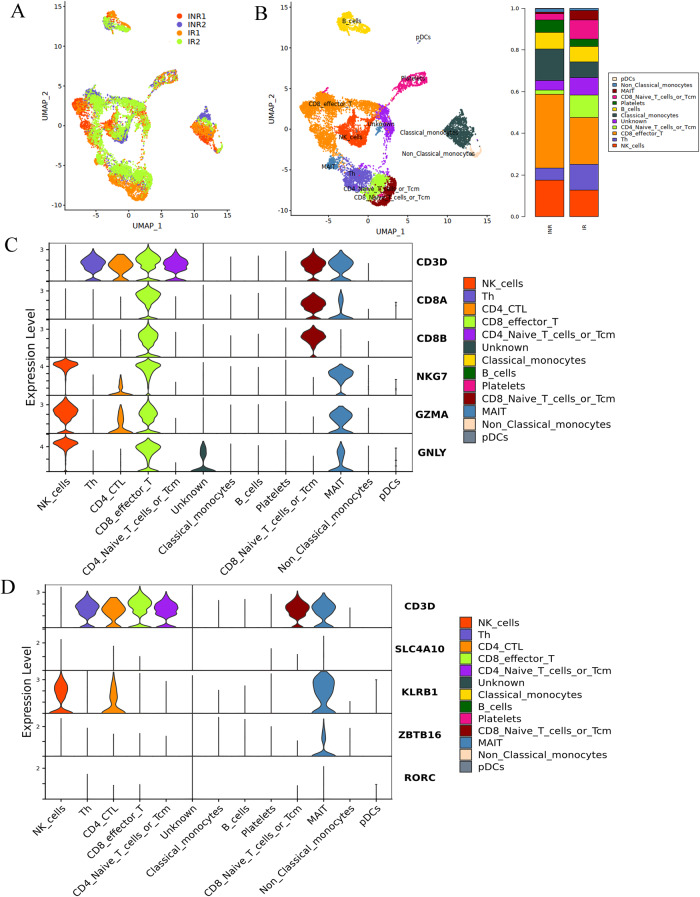


The original article has been corrected.

