# Supplementary material for: Single-cell sequencing resolves the landscape of immune cells and regulatory mechanisms in HIV-infected immune non-responders
Source: Cell Death Dis. 2022 Oct 4;13(10):849. doi: 10.1038/s41419-022-05225-6 (PMC9532384; doi:10.1038/s41419-022-05225-6)
Supplement: Supplementary file 2 — supplementary [file 41419_2022_5225_MOESM2_ESM.pdf]

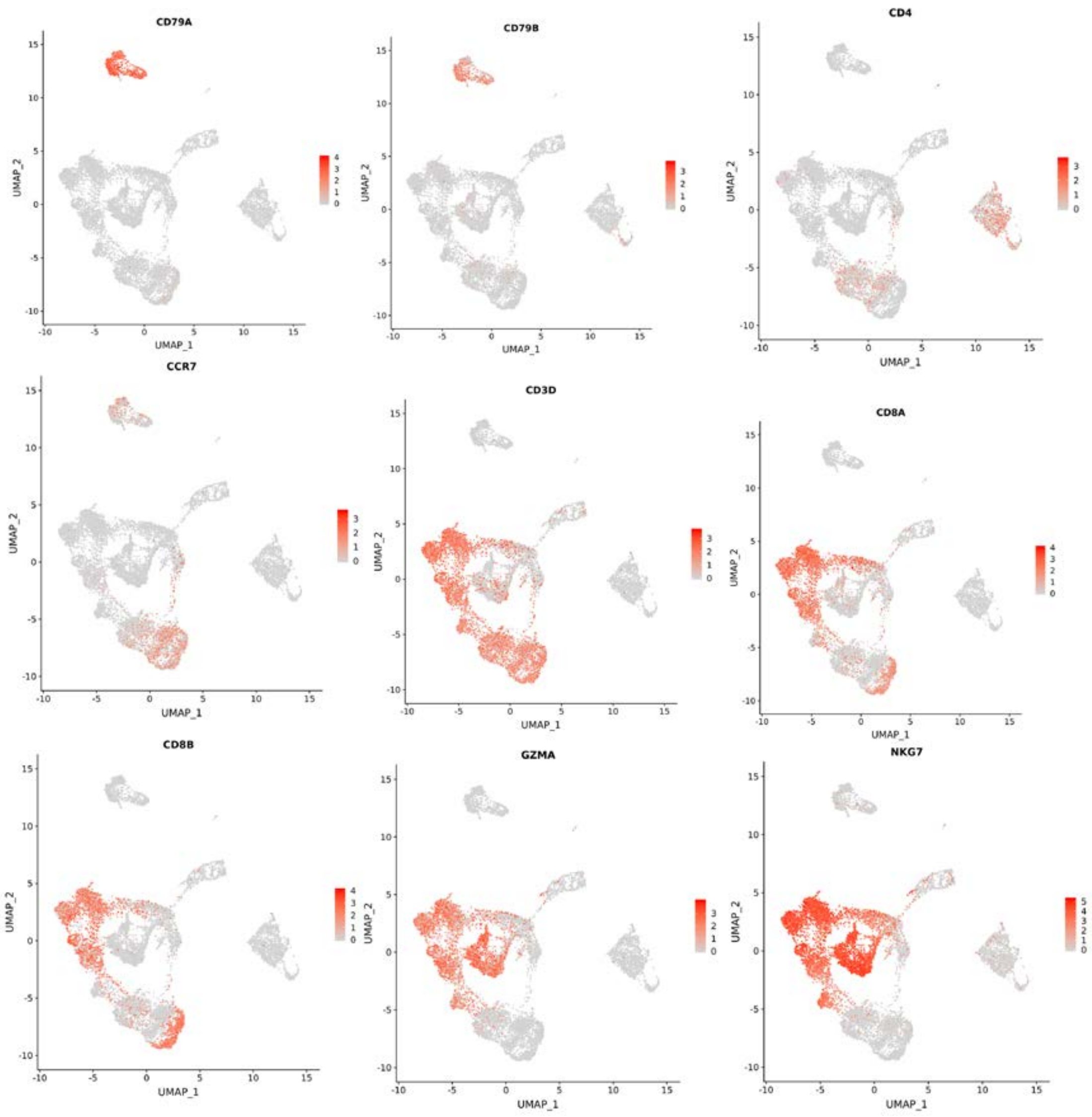

Supplementary figure Legends

Supplementary figure1 Projection of cells expressing the chosen transcripts to the UMAP plots.

Supplementary figure2 The open state of chromatin in INR was detected by scATAC-seq. (A) Analysis of gene chromatin transcription of immune cells in INR (B) Expression and motif of transcription factors RUNX3 and TBX21 in INR

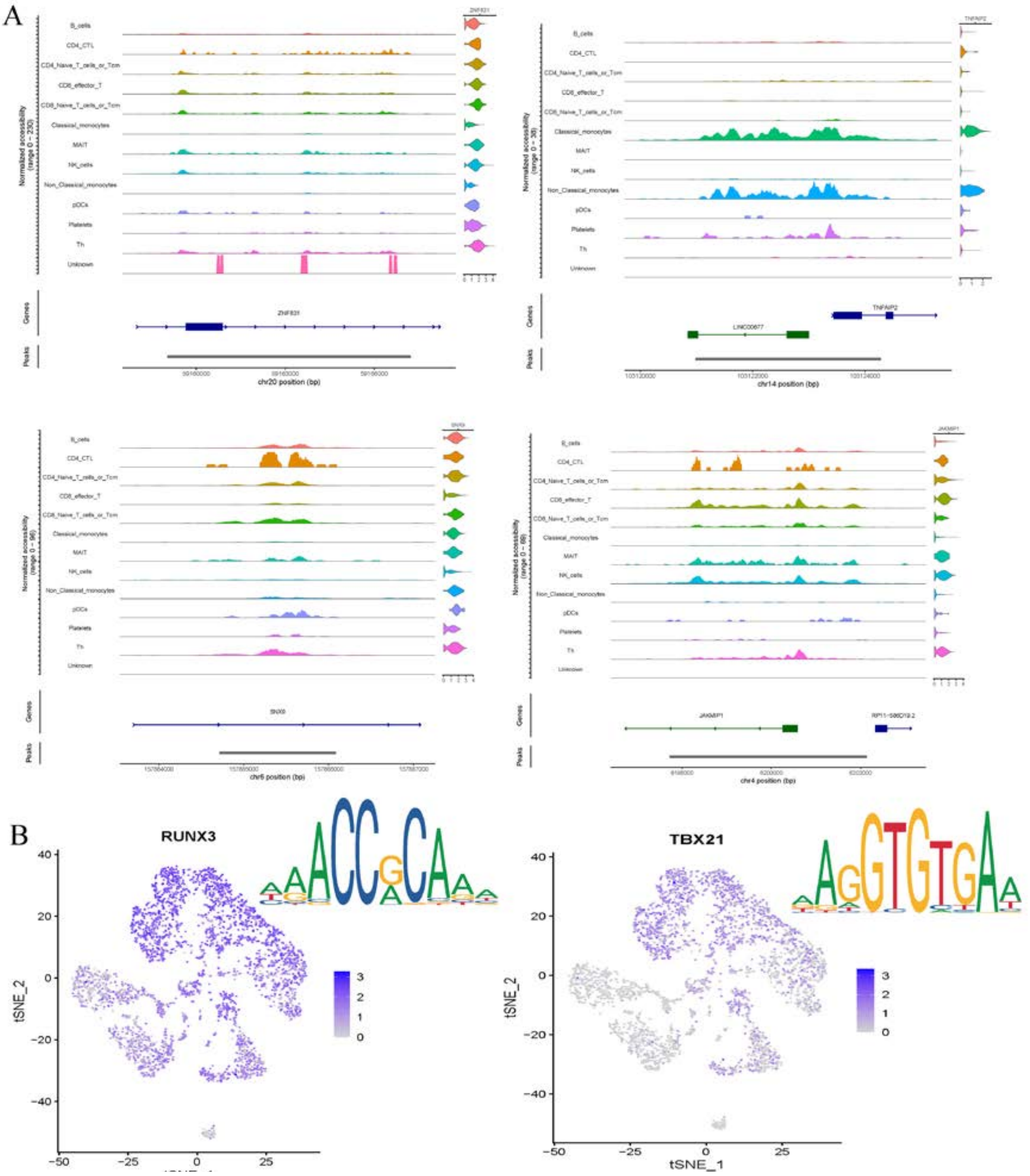
A
B_cells
CD4_CTL
CD4_Naive_T_cells_or_Tcm
CD8_effector_T
CD8_Naive_T_cells_or_Tcm
Classical_monocytes
MAIT
NK_cells
Non_Classical_monocytes
pDCs
Platelets
Th
Unknown
Genes
Peaks
ZNF831
chr20 position (bp)
TNFAIP2
LINC00677
chr14 position (bp)
SNX9
chr6 position (bp)
JAKMIP1
chr4 position (bp)
B
RUNX3
TBX21
tSNE_2
tSNE_1
