# Supplementary material for: Single-cell sequencing resolves the landscape of immune cells and regulatory mechanisms in HIV-infected immune non-responders
Source: Cell Death Dis. 2022 Oct 4;13(10):849. doi: 10.1038/s41419-022-05225-6 (PMC9532384; doi:10.1038/s41419-022-05225-6)
Supplement: Supplementary file 3 — supplementary Table 1 [file 41419_2022_5225_MOESM3_ESM.docx]

Supplementary Table 1. Cell type / abbreviation / cell type identification marker gene

| **cell type** | **abbreviation** | **marker gene** |
| --- | --- | --- |
| CD8+ Naïve T cells | CD8_Naïve T cells | CCR7,LEF1,SELL,TCF7,CD8A,CD8B |
| CD4+ Naïve T cells | CD4_Naïve T cells | CCR7,LEF1,SELL,TCF7,CD4 |
| T helper cells | Th | CD4,GATA3,ICOS,CD40LG,FOXP3 |
| CD8+ effector T cells | CD8_effector T | CD3D,CD8A/B,NKG7,GZMA,GNLY |
| Mucosal-associated invariant T cells | MAIT | CD3D,SLC4A10,KLRB1,ZBTB16,RORC |
| B cells | B cells | MS4A1,CD79A,CD79B |
| NK cells | NK cells | CD3D,KLRD1,NKG7,KLRF1 |
| Plasmacytoid dendritic cells | pDCs | IL3RA,CLEC4C,LILRB4 |
| Classical monocytes | Classical monocytes | LYZ,CD14,FCN1,VCAN |
| Non-Classical monocytes | Non-Classical monocytes | LYZ,FCGR3A,IFITM3 |
| Platelets | Platelets | PF4,PPBP,TUBB1 |
